# Aberrant SPOP-CHAF1A ubiquitination axis triggers tumor autophagy that endows a therapeutical vulnerability in diffuse large B cell lymphoma

**DOI:** 10.1186/s12967-022-03476-7

**Published:** 2022-06-30

**Authors:** Wei Yan, Xue Shi, Huihan Wang, Aijun Liao, Wei Yang

**Affiliations:** Department of Hematology, Shengjing Hospital of China Medical University, 39 Huaxiang Road, Shenyang, Liaoning China

**Keywords:** CHAF1A, SPOP, Ubiquitination, Autophagy, DLBCL

## Abstract

**Purpose:**

Aberrant epigenetic changes, like DNA methylation, histone modifications, or ubiquitination, could trigger metabolic disorders in human cancer cells. This study planed to uncover the biological roles of epigenetic SPOP/CHAF1A axis in modulating tumor autophagy during Diffuse large B-cell lymphoma (DLBCL) tumorigenesis.

**Materials and methods:**

The Immunohistochemistry (IHC) was performed to assess the CHAF1A expressions. The expression data of CHAF1A was derived from The Cancer Genome Atlas (TCGA), GSE32918 and GSE83632 datasets. Bioinformatic assays contain differential analysis, functional enrichment analysis and Kaplan–Meier survival curve analysis. The colony generation assay, Transwell assay and CCK-8 assays were conducted for the in vitro assays. The in vivo ubiquitination assays were used to assess regulations of SPOP on CHAF1A. The Chromatin immunoprecipitation (ChIP) assays were used to uncover epigenetic regulations of CHAF1A on TFEB. The relevant DLBCL cells were subcutaneously injected to SCID beige mice to establish the xenograft models.

**Results:**

Bioinformatic results revealed that CHAF1A expressed highly in DLBCL that were validated in patients samples. Patients with high CHAF1A suffered from inferior prognosis with shorter survival months relative to those with low CHAF1A. High CHAF1A enhanced DLBCL aggressiveness, including cell proliferation, migration and in vivo growth. Mechanistically, E3 ubiquitin ligase SPOP binds to and induces the degradative ubiquitination of CHAF1A via recognizing a consensus SPOP-binding motif in CHAF1A. SPOP is down-regulated in DLBCL and habours two DLBCL-associated mutations. Deficient SPOP leads to accumulated CHAF1A proteins that promote malignant features of DLBCL. Subsequently, ChIP-qPCR assay revealed that CHAF1A directly binds to TFEB promoters to activate the expressions. High CHAF1A could enhance the transcriptional activity of TFEB and downstream genes. The SPOP/CHAF1A axis modulates TFEB-dependent transactivation to regulate the lysosomal biogenesis and autophagy. The in vivo models suggested that TFEB inhibition is effective to suppress growth of SPOP-deficient DLBCLs.

**Conclusions:**

CHAF1A is aberrantly elevated in SPOP-deficient DLBCL. The in‐depth mechanism understanding of SPOP/CHAF1A/TFEB axis endows novel targets for DLBCL treatment.

## Introduction

Lymphoma is a common hematological malignant tumor that originates in the lymph nodes or lymphoid tissue, which could be classcially divided into Hodgkin’s Lymphoma (HL) and Non-Hodgkin’s Lymphoma (NHL) [1, 2]. The authoritative epidemiology data indicates that the 2022 estimated new cases of lymphoma would be 89,010, resulting in approximately 21,170 death in the United States [3]. Of note, among all pathological subtypes of NHL, Diffuse large B cell lymphoma (DLBCL) ranks the first type and occupies nearly 40% of all diagnosed cases [4]. Like the other subtypes of lymphoid malignancies, DLBCL exihibits various heterogeneous features in regard to morphology, biological aggressiveness, as well as clinical presentation [5, 6]. The rituximab, cyclophosphamide, doxorubicin, vincristine, and prednisone (R-CHOP) is an acknowledged standard strategy for DLBCL, alleviating burden of more two-thirds of cases [7]. However, nearly 30% of DLBCL patients still have unsatisfactory effects and suffer from recurrent attacks due to ambiguous symptoms and unclear pathogenesis [8]. As reported, three scoring systems intergrating multiple clinical characteristics have been commonly adopted to determine prognosis of patients for 20 years, like the International Prognostic Index (IPI), revised IPI (R-IPI), and National Comprehensive Cancer Network IPI (NCCN-IPI) [9–11]. Nevertheless, the NCCN-IPI score is not completely effective to predict the clinical outcomes of DLBCL, implicating the existed tumor heterogeneity [12]. In recent years, high-throughput sequencing methods have identified different molecular subtypes of DLBCL, like germinal center B-cell-like (GCB) DLBCL and activated B-cell-like (ABC) DLBCL [13]. Differing from GC-like subtype, ABC-like DLBCL correlates with a worse prognosis, which is characterized by constitutively activated NF-kB signaling [5]. As a result, further investigations on molecular mechanisms of DLBCL is of great importance to optimize prognostic hazard stratification and endow novel therapeutical targets.

In recent decades, numerous researches indicate that chromatin remodelers and histone modifiers are reported to exert essential roles in multiple tumorigenesis and aggressiveness, including DLBCL [14–16]. Of note, as a highly conserved histone chaperone heterotrimer and containing p48, p60, and p150 subunits (CHAF1A), chromatin assembly factor-1 (CAF-1) participates in various biological events, especially responsible for chromatin structure restoration upon DNA repair [17]. As the key component of CAF-1, CHAF1A cooperates with other factors, like heterochromatin protein 1 (HP1), to modulate DNA mismatch repair, DNA replication process, as well as epigenetic regulation of related genes. Besides, CHAF1A could also regulate H3K9 trimethylation that influences expressions of key target genes related with proliferation, survival, and differentiation [18]. For instance, CHAF1A participates in a complex with methyl CpG DNA binding domain protein 1 (MDB1) and histone methyl transferase SETDB1. Intensive documents have implicated that CHAF1A is abnormally regulated or expressed in various malignancies, including neuroblastoma, prostate cancer, breast cancer, as well as hepatocellular carcinoma (HCC) [17, 19, 20]. Furthermore, dysregulation of CHAF1A correlates tightly with genomic instability and leads to high morbid risks of leukemia, lymphoma, or other solid tumors. However, no studies are currently reported to elucidate the underlying associations between CHAF1A and lymphoma, especially the DLBCL. Meanwhile, the potential mechanisms that contribute to abnormal CHAF1A expressions are still unidentified.

As one of the adaptor proteins of the CUL3–RBX1 E3 ubiquitin ligase complex (CRL3), Speckle-Type Poz Protein (SPOP) could selectively recruit substrates via its.

N-terminal MATH domain [21]. Next, the BTB and BACK domains of SPOP are mainly responsible for promoting oligomerization and interaction with CUL3. A list of substrates of SPOP were identified that are oncoproteins in multiple tumors, including BRD4, AR, GLI, SRC-3, Caprin1, and PD-L1 [22, 23]. Besides, SPOP could also mediate the nondegradative ubiquitination of p62 at residue K420 within the UBA domain to attenuate the Nrf2-mediated transcriptional activation of antioxidant genes [24]. SPOP was regarded to be a tumor suppressor in many cancers, like prostate cancer, colorectal cancer, breast cancer, and endometrial cancer (PMID: 31,771,591; 31,772,275; 31,911,863). In contrast, the tumorigenic activity of SPOP in renal cell carcinoma occurs via the ubiquitination and degradation of many factors of cellular proliferation and apoptosis, like PTEN, ERK phosphatases, Daxx, and the Hedgehog pathway transcription factor Gli2 [25, 26]. These findings supported that SPOP may have double-faced and distinct roles in different tumors. However, little researches were available to uncover the associations between SPOP and lymphoma. Although Xiaofeng Jin et al. have found that CRL3–SPOP ubiquitin ligase complex suppresses the growth of diffuse large B-cell lymphoma by negatively regulating the MyD88/NF-κB signaling, whether SPOP plays essential functions in hematologic malignancies still remains undefinited [27]. Apart from NF-κB signaling, whether SPOP regulates other biological pathways involved in aggressiveness of DLBCL is an interesting project to be thoroughly elucidated.

In this study, we found CHAF1A is highly expressed in DLBCL and contributes to malignant proliferation and growth. SPOP functions as a negative regulator of CHAF1A via interacting with and inducing the degradative ubiquitination of CHAF1A. Down-regulated or DLBCL-associated SPOP mutations contribute to CHAF1A accumulations, thereby enhancing tumor autophagy of DLBCL in a TFEB-dependent manner. Therefore, our study provided a novel link between SPOP/CHAF1A axis and tumor autophagy of DLBCL, acting as the basis for finding novel epigenetic targets for DLBCL treatment.

## Methods and materials

### Cell culture

Human lymphoblastoid B cell (GM12878), human renal epithelial cells (293 T) and DLBCL cells (OCI-LY7, DB, U2932, and FARAGE) were purchased from American Type Culture Collection (ATCC, USA). The 293 T cells were maintained in DMEM with 10%(v/v) FBS. The GM12878 cells were cultured in Roswell Park Memorial Institute 1640 medium (RPMI 1640; Gibco) supplemented with 15% fetal bovine serum (FBS; Gibco) and 1% penicillin–streptomycin (Gibco) at 37℃ with 5% CO2.

### Collection of DLBCL tissues and immunohistochemistry (IHC) assays

Eighty paired B cell lymphoma tissues and adjacent normal tissues were obtained from the Shengjing Hospital of China Medical University. This study was reviewed and approved by the Shengjing Hospital of China Medical University. All participants had signed informed consent. When we obtained the samples, the tissues were frozen at − 80 °C. No patients had received chemotherapy or radiotherapy before surgery. For the IHC assay, DLBCL tissues from patients were taken to paraffin imbedding and cut, and stained by hematoxylin. 4 μm thick sections of tissues were sliced. After deparaffinization and rehydration, samples were blocked from endogenous peroxidases with 3% solution of hydrogen peroxide. Following this, IHC staining was performed using the specific primary antibodies against CHAF1A according to standard protocols. After 1 × PBS rinses for 15 min, tissue sections were incubated with the rabbit anti-goat biotinylated secondary antibody, and then followed by incubation with strept avidin-horseradish peroxidase complex (SABC) and stained with 3,3′-diaminobenzidine tetrachlorhydrate dihydrate (DAB). Sections were counterstained with hematoxylin. The staining results were evaluated by two independent observers.

### Quantitative real-time PCR (RT-qPCR)

Total RNA was isolated from cells using the TRIzol reagent (Tiangen), and cDNA was reversed-transcribed using the Superscript RT kit (TOYOBO) following the manufacturer’s instructions. PCR amplification was performed using the SYBR Green PCR master mix Kit (TOYOBO). All quantitations were normalized to the level of endogenous control GAPDH. The primers of genes in this study were listed as the following: CHAF1A: F: 5′-GATGCTGCGGAGGTCCAA-3′; R: 5′-ATACGTCACCCCTGCTCTCA-3′; TFEB: F: 5′-CAAGCTCAGGCTGGGAGC-3′; R: 5′-GTATTGATGGCCGGGGTGG-3′.

### CCK-8 and colony formation assays

First of all, the DLBCL cells (U2932, FARAGE) were seeded into 96-well plates with the concentration of 1 × 10^3^ cells/well. All cells were incubated for 0, 24, 48, 72, and 96 h. Next, the cells were subjected to the addition of cell counting kit 8 (CCK-8; Dojindo, Tokyo, Japan) and incubated for 2 h. The optical density was measured at 450 nm. For colony formation assay, indicated cells in 6-well plates (5 × 10^2^ cells/well) were cultured for two weeks. Then, the cells were subjected to the fixation using methanol and then stained using crystal violet (SigmaAldrich). The number of colonies containing more than 50 cells was counted manually.

### Transwell assays

Cell migration was determined by Transwell (Costar) migration assay. DLBCL cells (U2932, FARAGE) were precultured in serumfree medium for 48 h. For migration assay, 3 × 10^4^ cells were seeded in serum-free medium in the upper chamber, and the lower chamber was filled with RPMI1640 containing 5% FBS. After 48 h, the non-migrating cells on the upper chambers were carefully removed with a cotton swab, and migrated cells underside of the filter stained and counted in nine different fields.

### In vivo ubiquitination assays

The 293 T cells were transfected with HA-Ub and other indicated constructs. After 36 h transfection, cells were lysed in 1% SDS buffer (Tris pH 7.5, 0.5 mM EDTA, 1 mM DTT) and boiled for 10 min. For immunoprecipitation, the cell lysates were diluted tenfold in Tris–HCl buffer and incubated with anti-FLAG M2 agarose beads for 4 h at 4 °C. The bound beads are then washed four times with BC100 buffer (20 mM Tris–Cl, pH 7.9, 100 mM NaCl, 0.2 mM EDTA, 20% glycerol) containing 0.2% Triton X-100. The protein was eluted with 3 × FLAG peptide for 2 h at 4 °C. The ubiquitinated form of CHAF1A was detected by Western blot using the anti-HA antibody.

### Western blotting

After 48 h transfection, cells were lysed by RIPA lysis buffer (Beyotime, Shanghai, China) supplemented with protease inhibitor PMSF (Wuhan Boster Biological Technology, Ltd, Wuhan, China). Protein concentration was detected with bicinchoninic acid (BCA) method. Separated proteins were denatured at 95 °C for 5 min. Equal amount of protein (20 μg) was added into each well in 12% SDSPAGE, and transferred onto PVDF membranes (Millipore, Billerica, MA, USA). Afterwards, PVDF membranes were blocked in 5% skim milk for 1 h at room temperature, following the incubation with specific primary antibodies at 4 °C overnight. PVDF membranes were then sealed with secondary antibody for 1 h at 37 °C. The protein signals were visualized with ECL solution (Millipore) and scanned by QUANTITY ONE software (Bio-Rad, Hercules, CA, USA). All antibodies used in this study were listed as the following: anti-SPOP (Abcam, ab192233); anti-CHAF1A (Cell signaling Technology, CST#5480); anti-HA (Abcam, ab9110); anti-p62 (Cell signaling Technology, CST#23,214); anti-Beclin-1 (Cell signaling Technology, CST#4122); anti-β-actin (Cell signaling Technology, CST#41,470); anti-FLAG (Cell signaling Technology, CST#14,793).

### Chromatin immunoprecipitation (ChIP)

ChIP assay was conducted by the application of SimpleChIP® Enzymatic Chromatin IP Kit (Magnetic Beads) #9003 (Cell Signaling Technology, USA) in accordance with the manufacturer's instructions. Antibodies against CHAF1A was applied to mmunoprecipitate the crosslinked protein-DNA complex, with anti-IgG as negative control. The immunoprecipitated DNA underwent purification and was analyzed by RTqPCR with primers specific for the predicted binding sites on the promoter of TFEB.

### Luciferase reporter assay

The pmirGLO dual-luciferase vector (Promega, Madison, WI, USA) containing TFEB sequence was cotransfected with CHAF1A plasmids into 293 T cells. The luciferase reporter assay was conducted in shCtrl and shCHAF1A#1/2 cells. The TFEB promoter was sub-cloned into the pGL3-basic vector (Promega), then co-transfected into 293 T cells with EV or FLAG-CHAF1A, individually. Luciferase activities were explored via DualLuciferase Reporter Assay System (Promega).

### Transmission electron microscopy (TEM)

DLBCL cells were fixed with 2.5% glutaraldehyde and 1% osmium tetroxide at 4 °C and immersed in spur resin after dehydration. The cells were then stained with 4% uranyl acetate and lead citrate. Finally, images were captured using a TEM (Hitachi, Ltd).

### In vivo tumor model

The 6-week-old beige female mice with severe combined immunodeficiency (SCID) were obtained from the SLAC Laboratory Animal Co., Ltd. and reared in a pathogen-free environment. All mice were randomly divided into two groups. As previously illustrated, DLBCL cells (either transfected with CHAF1A-OE vectors or empty control vectors) were subcutaneously injected to SCID-beige mice to establish xenograft models (n = 8 per group). At the end of 5 weeks, mice were killed and in vivo tumors were dissected and weighed. The ethics approval number for animal assays is 20210014-R.

### Confocal microscopy

The sections were fixed and permeabilized with 0.3% Triton (Solarbio Life Sciences) for 10 min. After blocking non-specific binding using 5% BSA, sections were incubated with LC3 (1:100, Cat No. 14600–1-AP, Proteintech Group, Inc). After transfecting RFP-GFP-LC3 adenovirus for 48 h, DLBCL cells were fixed, and autophagosome dots were photographed and counted using a laser scanning confocal microscope.

### Statistical analysis

Data were revealed as mean ± SD. Variance analyses were implemented via Student’s *t* test or one-way ANOVA. Pearson Correlation Coefficient was utilized for verifying significance of the correlation among SPOP, CHAF1A and TFEB expression. The *P* < 0.05 was considered statistically significant. Statistical analyses were conducted employing SPSS 22.0 (IBM, Armonk, NY, USA). All experiments were repeated in independent experiments at least three times.

## Results

### High CHAF1A in DLBCL is a prognostic factor and correlates with poor prognosis

**Fig. 1:**
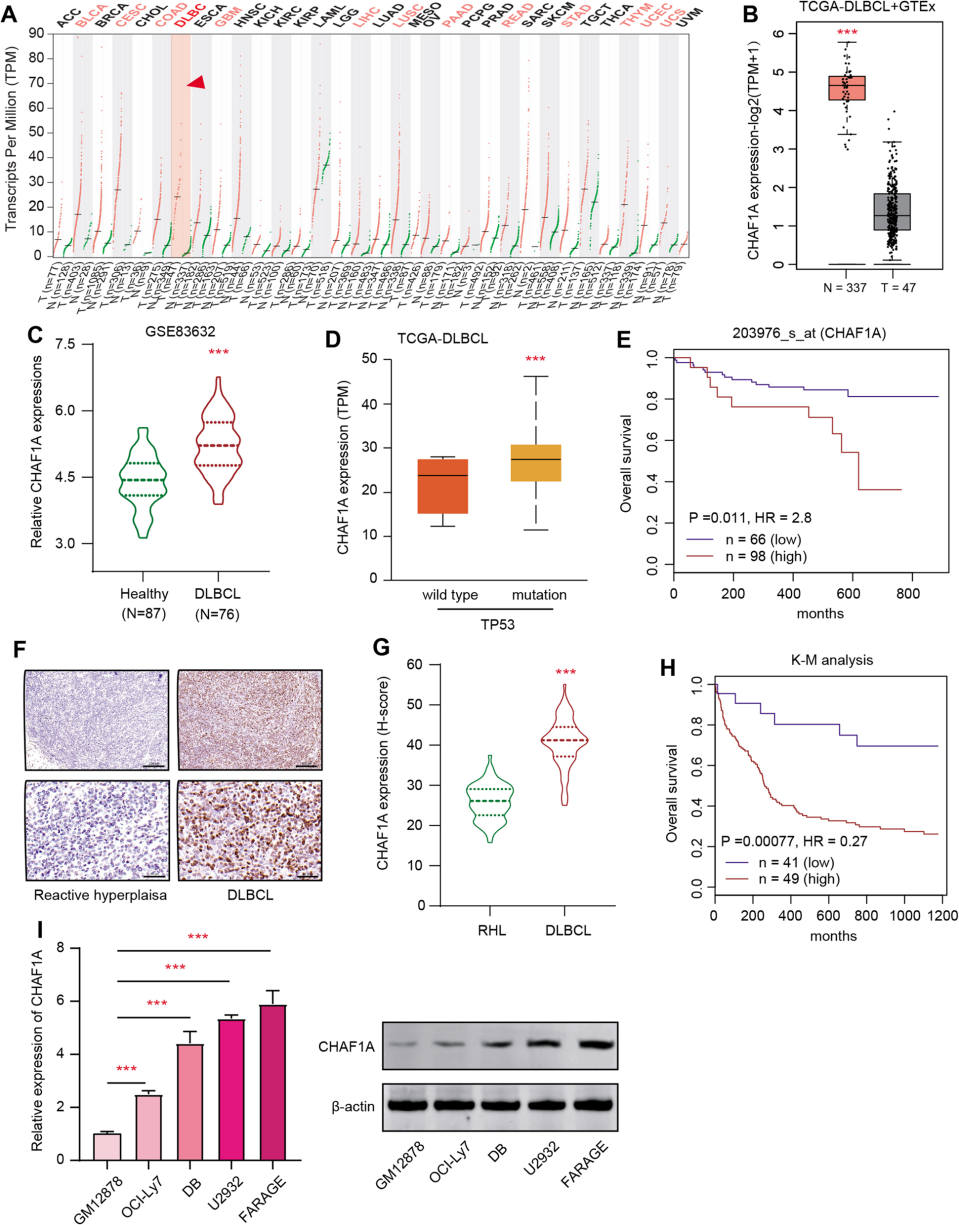
CHAF1A is highly expressed in DLBCL compared with benign healthy tissues. **A** Pan-cancer profiles based on GEPIA2 platform showing the expression levels of CHAF1A in multiple malignancies matched with respective normal samples, individually. **B** Differential analysis based on TCGA-DLBCL cohort revealing that CHAF1A was elevated in DLBCL (N = 47) versus GTEx normal tissues. **C** Besides, CHAF1A was up-regulated in DLBCL (N = 164) as compared to normal samples in the GSE83632 dataset. **D** Based on TCGA-DLBCL samples, TP53-mutated samples exhibit high CHAF1A expressions. **E** Prognostic analysis with Kaplan–Meier survival curves indicated that DLBCL samples with high CHAF1A shorter overall survival months relative to those with low CHAF1A expressions based on the GSE32918 data set (log-rank test p = 0.011). **F** As evidenced by IHC assay, CHAF1A expressions were notably higher in DLBCL tissues versus RHL. Upper scale bar = 200 μm, lower scale bar = 50 μm. **G** Differential test analysis of quantified CHAF1A expressions (*h-score*) in collected DLBCL and RHL samples (N = 70). **H** High CHAF1A expression in DLBCL correlated with shorter overall survival months based on the analysis of the IHC levels, as revealed by the Kaplan–Meier survival curve analysis (n = 90, p < 0.001). **I** The RT-qPCR analysis indicated the upregulated expression of CHAF1A in multiple DLBCL cell lines. Experiments were performed in triplicate. *p < 0.05, **p < 0.01, ***p < 0.001

To assess the potential significance of CHAF1A in DLBCL, we firstly explored the expressions of CHAF1A in 33 pan-cancer samples matched with individual normal tissues based on the GEPIA2 platform (http://gepia2.cancer-pku.cn/#index). In line with the findings in BLCA, CESC, or STAD, CHAF1A is also aberrantly elevated in DLBCL compared with paired normal samples (Fig. 1A, B). Besides, we queried the expression data derived from GSE83632 (N = 163) in GEO database and observed that CHAF1A was notably upregulated in DLBCL versus normal samples based on differential analysis (Fig. 1C). Meanwhile, we categorized the TCGA-DLBCL samples into TP53 mutation and wild type samples, and uncovered that high CHAF1A correlated with TP53 alterations (Fig. 1D). Based on the DLBCL samples (N = 249) in GSE32918 from the GEO database, we conducted the Kaplan–Meier survival curve analysis and found that high CHAF1A levels correlated with shorter overall survival (OS) months in contrast to those patients with low CHAF1A (log-rank test *P* = 0.011, Fig. 1E). Accordingly, we collected 70 paired DLBC samples in our center and noted the elevated CHAF1A expressions in DLBCL tissues relative to tissues with RHL based on the Immunohistochemistry (IHC) method (Fig. 1F, G). Kaplan-Meir analysis also indicated that higher CHAF1A expression in DLBCL was associated with shorter OS months (log-rank test *P* < 0.0001, N = 90, Fig. 1H). Lastly, compared with control cells obtained from healthy volunteers, CHAF1A proteins were markedly higher in DLBCL cell lines (Fig. 1I). In summary, these results indicated that high CHAF1A expressions have clinical significance in DLBCL.

### CHAF1A pomotes DLBC malignant growth in vitro and in vivo

**Fig. 2:**
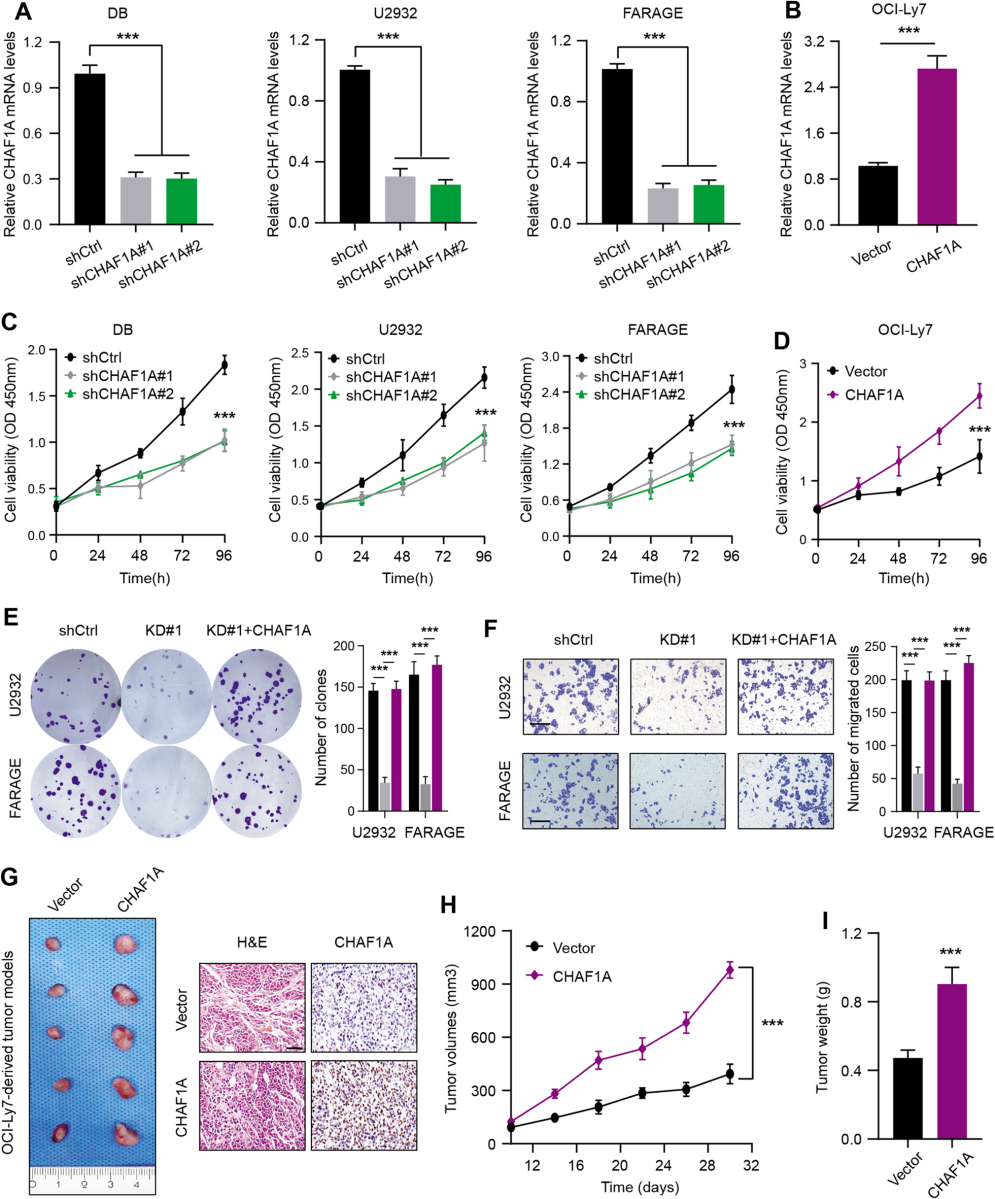
High CHAF1A exerts tumor-promoting biological effects in DLBCL. **A** The RT-qPCR analysis indicated the down-regulated CHAF1A mRNA expressions shCHAF1A DLBCL cell lines (DB, U2932 and FARAGE) and shCtrl cells. **B** The RT-qPCR analysis indicated the elevated CHAF1A expressions in EV and OE OCI-Ly7 cells. **C** MTT assay showed suppressed cell viability when CHAF1A was knocked down by shCHAF1A in three independent DLBCL cell lines as compared to control (shCtrl) cells. **D** In contrast, CHAF1A overexpression could cellular proliferative activity of OCI-Ly7. **E** The colony generation of DLBCL cells by colony formation assays showed CHAF1A KD could attenuate colony formation ability, but CHAF1A OE could rescue the suppressed capacity. **F** Besides, CHAF1A KD could also attenuate migration ability of cells by Transwell assay, but CHAF1A OE could also rescue the impaired migration capacity. **G** Animal assays on nude mice with OCI-Ly7 cells showed enhanced tumor growth rate in the CHAF1A OE group compared with the control group (EV). Representative tumor graphs were shown on the left, while H&E and IHC staining of Ki-67 were exhibited on the right. Scale bar = 50 μm **H** Record of tumor volumes at the indicated timepoints and generation of tumor growth curves in two mice groups. **I** After the animal sacrifice, tumor weight of mice derived from the two groups were compared. Experiments were performed in triplicate. *p < 0.05, **p < 0.01, ***p < 0.001

To validate whether CHAF1A exerts a biological impact in DLBCL, we performed the gain- and loss-of-function assays using lentivirus infection technology. Two independent lentivirus mediated RNA interference (RNAi) vectors carrying GFP against CHAF1A demonstrated the effective knockdown efficacy of CHAF1A in DB, U2932, and FARAGE cells (Fig. 2A). The qRT-PCR experiments further showed the overexpression of CHAF1A in DLBCL cells via infection (Fig. 2B). Firstly, CHAF1A knockdown remarkably suppressed the proliferative capacity of DLBCL cell lines, as indicated by the CCK-8 assays (Fig. 2C). In contrast, stable CHAF1A overexpression enhanced cell growth (Fig. 2D). Subsequently, depletion of CHAF1A weakened the cell viability, colony formation ability and invasive efficacy of two DLBCL cell lines, including U2932 and FARAGE (Fig. 2E, F). However, ectopic expression of CHAF1A completely rescued the impaired colony formation and invasive functions of cells (Fig. 2E, F). Lastly, we constructed the mouse xenograft model using human DLBCL cells to confirm the in vivo functions of CHAF1A. The subcutaneous injections of either control or CHAF1A-overexpressing OCI-Ly7 cells were conducted on SCID mice (N = 8 per group). Notably, in line with in vitro results, mice with overexpressing CHAF1A suffered from more serious tumor burden and higher Ki-67 staining levels relative to those in the control group (Fig. 2G–I). Taken together, it was suggested that high CHAF1A served as an oncogene in DLBCL by enhancing cell proliferation and migration in vitro and in vivo.

### Aberrant SPOP-CHAF1A ubiquitination axis contributes to high CHAF1A expressions

**Fig. 3:**
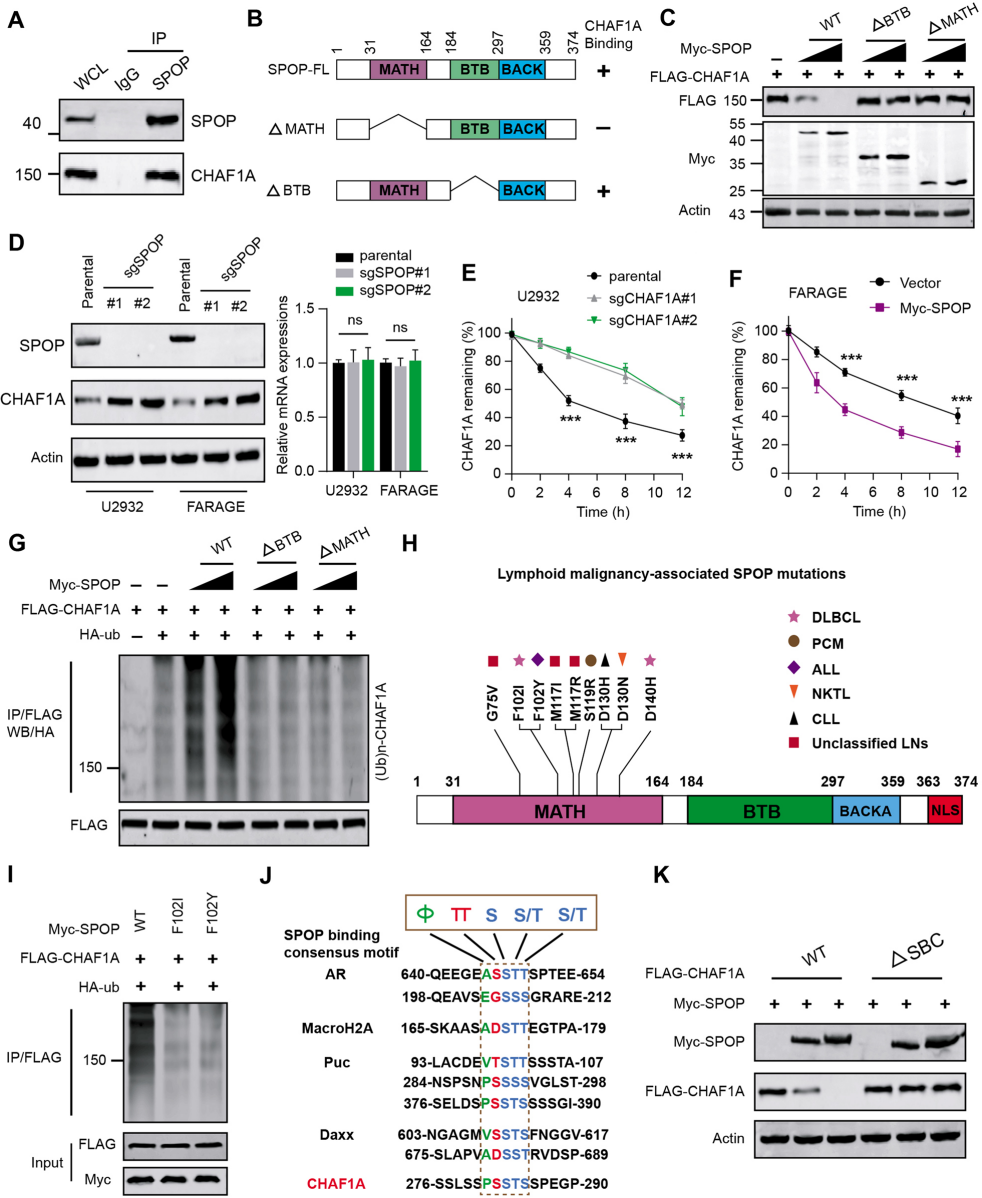
Aberrant SPOP-CHAF1A ubiquitination axis contributes to accumulated CHAF1A expressions in DLBCL. **A** Co-Immunoprecipitation (IP) analysis based on U2932 cells with IgG or anti-SPOP antibody indicated the endogeous interactions between SPOP and CHAF1A, as shown by western blotting assay. **B** The schematic representation of SPOP-WT and deletion mutants. Binding capacity of SPOP to CHAF1A is indicated with the representative symbol. **C** Western blotting assay showed the altered CHAF1A proteins in whole cell lysate (WCLs) from U2932 cells which were co-transfected with plasmids of FLAG-CHAF1A and WT SPOP or mutants. **D** Western blotting analysis showed the elevated CHAF1A proteins in CHAF1A KD cells (U2932, FARAGE) relative to control cells. Besides, the corresponding mRNA levels of CHAF1A in indicated groups were quantified and shown on the right, and no altereations were observed. **E** Quantification of CHAF1A proteins in WCLs of U2932 cells (parental, sgSPOP) for 12 h and then treated with 50 μg/ml cycloheximide (CHX) and harvested at different time points. At each time point, the intensity of CHAF1A was normalized to the intensity of actin and then to the data at 0 h. **F** Quantification of CHAF1A proteins in WCLs of FARAGE cells (EV, myc-SPOP) for 12 h and then treated with 50 μg/ml cycloheximide (CHX) and harvested at different time points. **G** After the 293 T cells were transfected with the indicated plasmids and treated with 20 μM MG132 for 8 h, the western blotting assay showed the in vivo CHAF1A ubiquitination levels by WT SPOP or mutants. **H** Illustration of tumor-associated mutations across SPOP gene in lymphoid malignancies. DLBCL: diffuse large B Cell lymphoma; PCM: plasma cell myeloma; ALL: acute lymphoid leukemia; NKTL: NK-T cell lymphoma; CLL: chronic lymphocytic leukemia; unclassified LNs unclassified lymphoid malignancies. **I** Western blotting assay showed the differential in vivo ubiquitination levels of CHAF1A in 293 T cells transfected with plasmids of FLAG-CHAF1A and Myc-SPOP or relevant mutations. **J** Comparison of putative SPOP binding sites in CHAF1A with the SPOP-binding consensus motif defined in the known SPOP substrates, where amino acid sequence alignment of the SBC motif (Φ-π-S-S/T-S/T; Φ: nonpolar residues, π: polar residues) was identified. **K** Western blotting assay showed the altered protein levels of WT-CHAF1A and mutants with deleted SBC motif when U2932 cells were transfected with Myc-SPOP, respectively. Experiments were performed in triplicate. *p < 0.05, **p < 0.01, ***p < 0.001

During the previous investigations, we screened that CHAF1A may interact with SPOP based on the BioGRID (Version 4.4) platform (https://thebiogrid.org/) [28, 29]. We thus wondered whether SPOP alterations may contribute to abnormal CHAF1A expressions in DLBCL. To verify that CHAF1A is a bona fide SPOP interacting protein, we carried out the Co-Immunoprecipitation (Co-IP) analysis with the anti-SPOP antibody, in which we found endogenous SPOP immunoprecipitated CHAF1A (Fig. 3A). As is well documentd, there were two structural domains in SPOP, containg substrate-binding MATH domain at the N-terminus and the CUL3-binding BTB domain at the C-terminus (Fig. 3B). We thus generated SPOP-ΔBTB and -ΔMATH mutants to find that only wild type SPOP could effectively decrease CHAF1A protein levels, not the other two mutants (Fig. 3C). We further deleted SPOP in U2932 and FARAGE cells to find that SPOP deletion signficantly promoted CHAF1A expressions, but not the mRNA levels (Fig. 3D). Knockout of SPOP markedly prolonged the half-life of CHAF1A protein in DB cells, as evidenced by the cycloheximide (CHX) assay (Fig. 3E). Conversely, SPOP overexpression by lentivirus infection significantly shortened the half-life of CHAF1A protein (Fig. 3F). Actually, CHAF1A proteins were robustly polyubiquitinated in a dose-dependent manner by the co-expression of SPOP-WT, but not by the SPOP-ΔBTB or ΔMATH mutant (Fig. 3G). Thus, these data suggest that CHAF1A is a bona fide of SPOP, and wild type SPOP regulates CHAF1A protein stability via ubiquitin-dependent proteasomal degradation in 293 T cells. Considering that whether SPOP contains defective mutations in lymphoid malignancies, we queried the data based on the cBioPortal (https://www.cbioportal.org/) and COSMIC (https://cancer.sanger.ac.uk/cosmic) to identify putative mutations of SPOP. Apparently, we obtained that SPOP was mutated in DLBCL, including F102I and D140H (Fig. 3H). Besides, we also found other mutations, like F102Y in acute lymphoid leukemia, S119R in plasma cell myeloma, D130H in NK-T cell lymphoma, D130N in chronic lymphocytic leukemia, and among others (Fig. 3H). SPOP-mediated CHAF1A ubiquitination was also notably attenuated with DLBCL mutants (F102I, D140H) in Fig. 3I. Since previous documents have indicated that one or several SPOP-binding consensus SBC motifs are present in SPOP substrates. Thus, we conducted a protein motif screen in this region and found a perfectly matched SBC motif (PSSTS) in CHAF1A-CDS, like the other substrates (Fig. 3J). We further generated the CHAF1A mutant in which the SBC motif sequence (PSSTS) was deleted. Intriguingly, wild type SPOP could promote CHAF1A ubiquitination and degradation, but not the mutant with deleted SBC motif, implicating that the SBC motif within CHAF1A is indispensable for SPOP-dependent ubiquitination and degradation (Fig. 3K). Taken together, DLBCL-related SPOP mutations could result in the stabilization of CHAF1A protein in DLBCL cells.

### Loss-of-function of SPOP depends on CHAF1A to amplify maligant features of DLBC

**Fig. 4:**
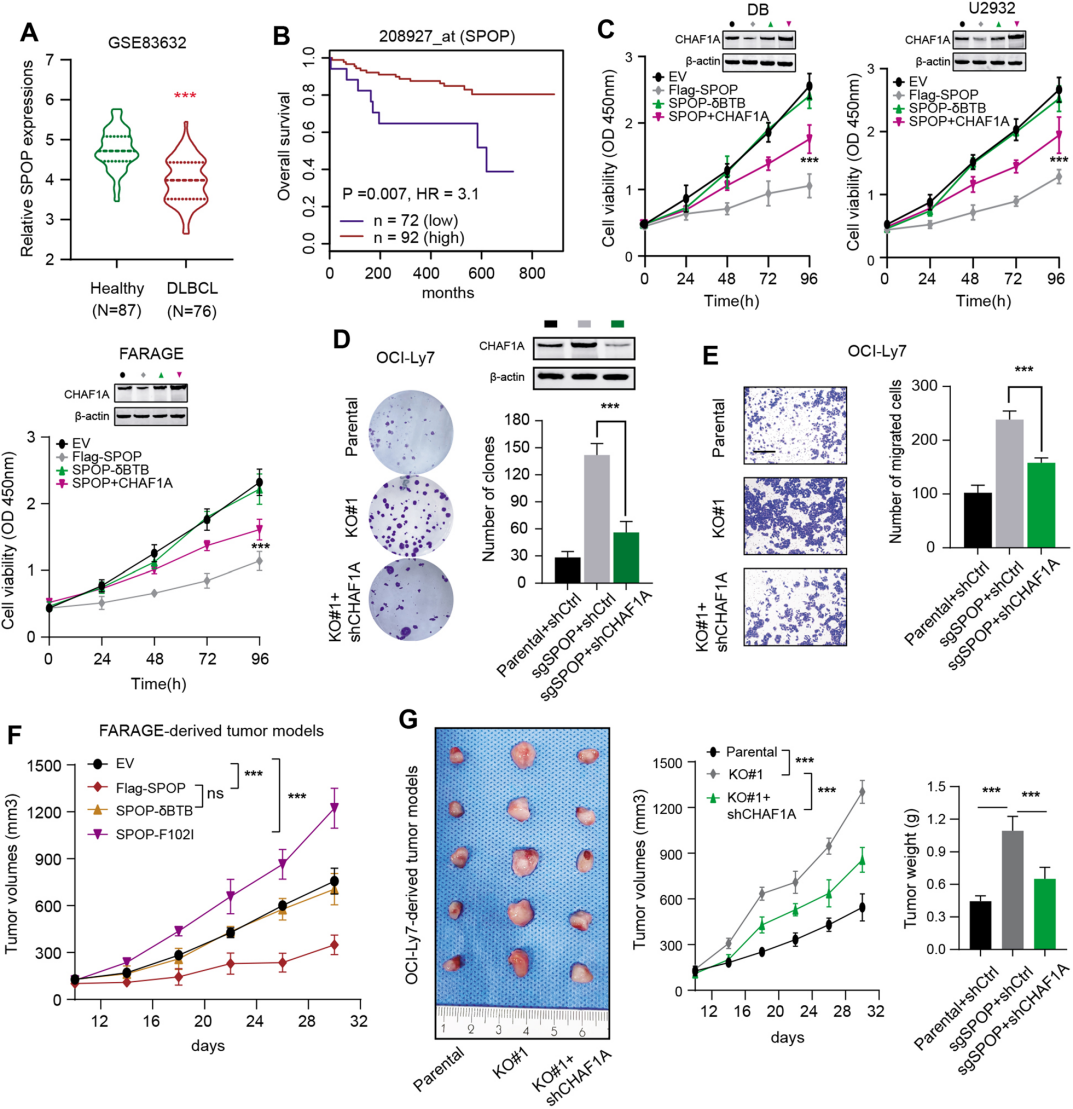
Down-regulated or mutated SPOP promotes DLBCL maliganant aggressiveness depending on CHAF1A. **A** The violin plot exhibited the differential expression levels of SPOP between DLBCL and normal tissues based on the GSE83632 data set. **B** Kaplan–Meier (K–M) survival curves analysis also showed that low SPOP levels may appear to correlate with shorter OS months of DLBCL patients based on the GSE83632 data set. **C** The CCK8 assays in three DLBCL cell lines (DB, U2932, FARAGE) revealed that WT SPOP, but not the δBTB mutant, could suppress cell proliferation, but simultaneous overexpression of CHAF1A could partially rescue the impaired cell growth ability. **D** Proliferation ability of OCI-Ly7 cells was enhanced by SPOP depletion, which could be partailly impaired by CHAF1A KD. **E** Similarly, SPOP depletion could enhance migration of OCI-Ly7 cells, which could be partially suppressed by CHAF1A KD. **F** Tumor volumes curves of FARAGE-derived tumor models showed that WT SPOP, but not the δBTB mutant, could suppress in vivo tumor growth, whereas tumor-associated SPOP F102I mutant could enhance in vivo tumor growth. **G** The OCI-Ly7-derived tumor models showed that SPOP depletion could enhance tumor in vivo growth, which could be partially suppressed by CHAF1A KD. Tumor graph was shown on the left; Quantification of tumor volumes was shown on the middle panel; Tumor weight was compared and shown on the right panel. Experiments were performed in triplicate. *p < 0.05, **p < 0.01, ***p < 0.001

We also detected the SPOP levels and found that SPOP expressed lowly in DLBCL versus normal tissues based on the GSE83632 data set (N = 164) in Fig. 4A. Kaplan–Meier analysis in GSE83632 data set also indicated that patients with low SPOP levels suffered from poor prognosis relative to those with high SPOP levels (Fig. 4B). We further found that SPOP depletion could remarkably enhance cell proliferation, colony formation ability and migration abilities (Fig. 4C–E). These data indicated that down-regulated SPOP in DLBCL indicated poor prognosis. We further utilized the lentivirus infection method to attenuate CHAF1A expressions in SPOP-deleted cells and found that CHAF1A knockdown significantly impaired the malignant features of SPOP-deficient DLBCL (Fig. 4C–E). Finally, the effect of SPOP/CHAF1A axis on DLBCL tumorigenesis was determined through animal models. The parental or SPOP-deleted OCI-Ly7 cells infected by lentivirus to knockdown CHAF1A were injected into the nude mice, individually. We observed that SPOP deficiency notably promoted DLBCL tumor growth in mice compared with tumors derived from parental OCI-Ly7 cells. However, targeting CHAF1A could abrogate the facilitative effect of SPOP loss on tumor growth, as indicated by tumor volumes and weight (Fig. 4F, G). Collectively, these results implicated that SPOP deficiency exerts oncogenic effects, which were at leat partially dependent of CHAF1A.

### SPOP-CHAF1A axis controls tumor autophagy that endows a therapeutical vulnerability

**Fig. 5:**
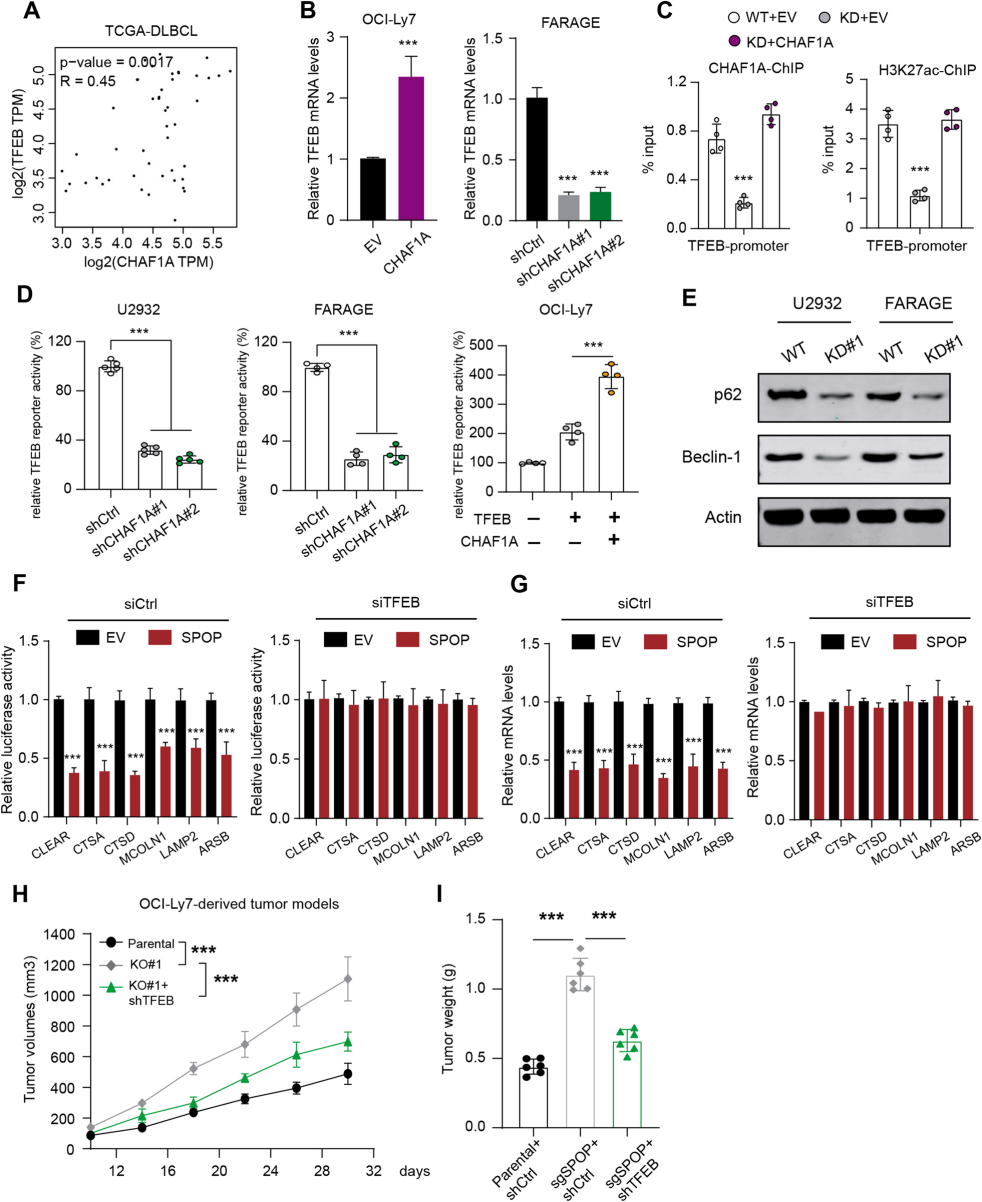
SPOP-CHAF1A axis controls tumor autophagy of DLBCL in a TFEB-dependent manner. **A** Positive associations between CHAF1A and TFEB expressions based on TCGA-DLBCL samples. **B** The RT-qPCR assay showed that CHAF1A OE could promote TFEB expression (left), while CHAF1A KD reduced TFEB mRNA levels (right panel). **C** The ChIP-qPCR of H3K27ac markers and CHAF1A in promoter of TFEB gene in U2932 cells, as indicated (N = 4). **D** The RT-qPCR assay showed that CHAF1A KD could suppress TFEB transcriptional activity in U2932 cells (left panel) and FARAGE cells (middle panel). Relative luciferase activities were normalized versus control. The OCI-Ly7 cells were transfected with TFEB reporters together with TFEB vectors alone or TFEB + CHAF1A plasmids to show the regulation of TFEB reporter activity by CHAF1A (right panel). **E** Western blotting assay showed that CHAF1A-KD decreased the levels of autophagy-related markers (p62, and Beclin-1) in CHAF1A-silenced U2932 and FARAGE cells relative to control cells. **F-G** The luciferase activity (**F**) and mRNA levels (**G**) of lysosomal genes were measured in control and SPOP-OE OCI-Ly7 cells transfected with siCtrl or siTFEB, individually. **H** The tumor growth curves were obtained from OCI-Ly7-derived tumor models to show that targeting TFEB significantly suppressed the enhanced tumor growth induced by SPOP deficiency. **I** The tumor weight of mice from indicated groups was shown and compared. Experiments were performed in triplicate. *p < 0.05, **p < 0.01, ***p < 0.001

**Fig. 6:**
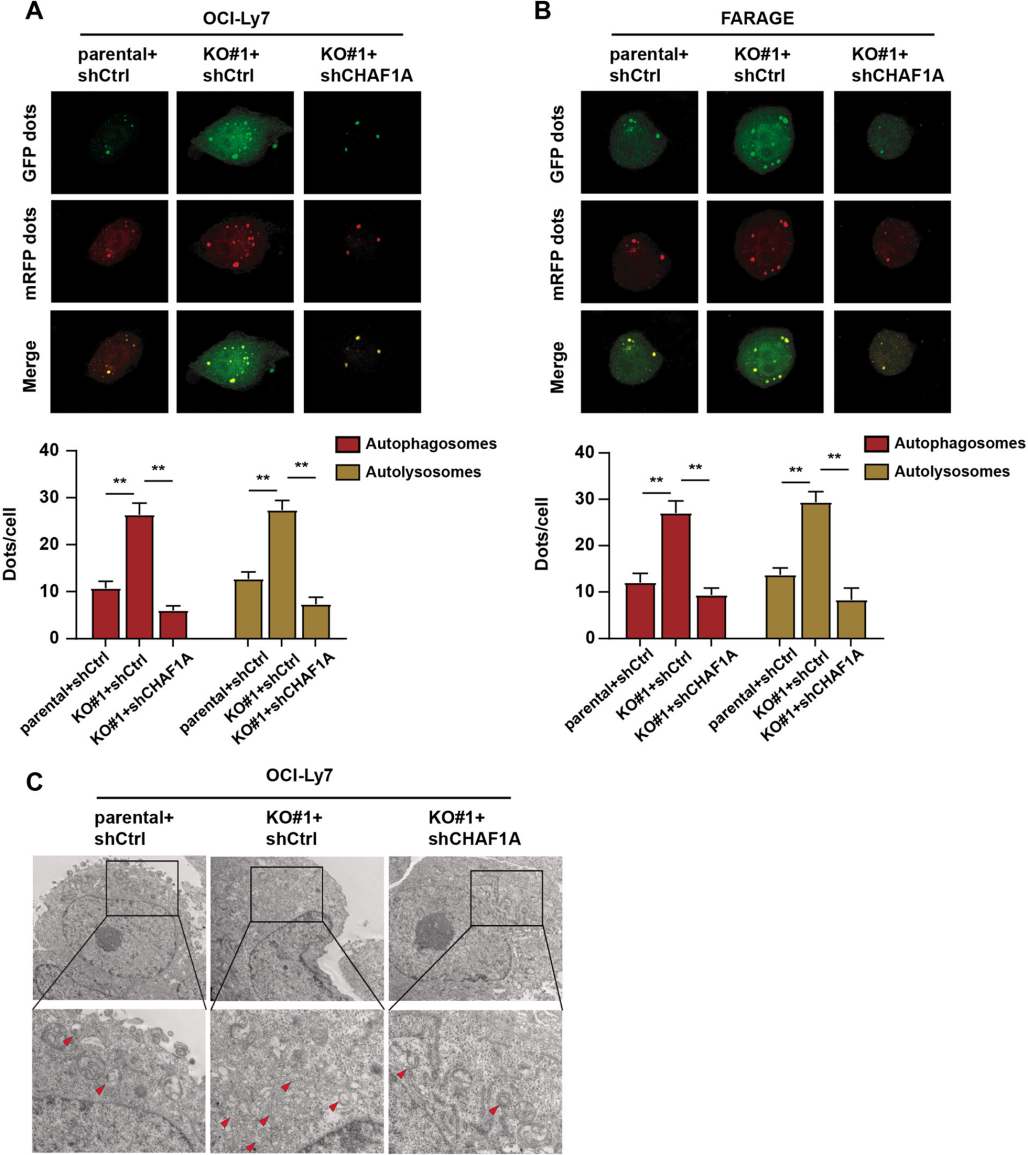
CHAF1A knockdown reversed the promotive effects of SPOP deficiency on DLBCL cell autophagy. **A**, **B** The confocal microscopy showed the autophagy flux in the indicated groups of DLBCL cells. **C** The TEM analysis showed the number of autophagic vacuoles in the indicated groups of DLBCL cells

When we explored the potential downstream signaling that SPOP/CHAF1A may rely on to mediate DLBCL tumorigenesis, we observed the positive relationships between CHAF1A and TFEB based on the TCGA-DLBCL data set (Fig. 5A). Besides, we used the RT-qPCR method to detect that overexpressing CHAF1A elevated TFEB levels, whereas CHAF1A knockdown suppressed the TFEB levels (Fig. 5B). The ChIP-qPCR assay further indicated that CHAF1A could directly bind to the promoter region of TFEB, along with the active H3K27ac modification indicator (Fig. 5C). Given that TFEB is the master transcription factor in the regulation of lysosomal biogenesis and autophagy, we thus wondered whether CHAF1A could modulate tumor autophagy process via TFEB activation. Expectedly, we observed that CHAF1A correlated positively with TFEB downstream lysosomal genes, like CLEAR, CTSA, or CTSD (Fig. 5D). Targeting CHAF1A significantly attenuated the levels of autophagy-related markers (p62, and Beclin-1) in CHAF1A-silenced U2932 and FARAGE cells, suggesting that CHAF1A could promote DLBCL autophagy (Fig. 5E). Given that previous studies showed that SPOP could restrict tumor autophagy via p62, we thus questioned whether SPOP could depend on CHAF1A/TFEB to regulate autophagy. Compared with vector control, SPOP could suppress transcriptional activity of TFEB by luciferase activities of lysosomal gene promoters in OCI-Ly7 cells, but TFEB knockdown reversed the inhibition (Fig. 5F). Accordingly, SPOP could restrict mRNA levels of TFEB downstream genes, which could be reversed by siTFEB (Fig. 5G). These data revealed that CHAF1A/TFEB aixs was indispensable.

for the inhibitory effects of SPOP on lysosomal biogenesis and autophagy in DLBCL cells. Based on these findings, we utilized control and SPOP-deleted OCI-Ly7 cells infected with shCtrl or shTFEB to generate the subcutaneous tumor models. In line with the findings in Fig. 4G, we found that targeting TFEB significantly suppressed the enhanced tumor growth induced by SPOP deficiency, as shown by tumor volumes (Fig. 5H). Given that SPOP/CHAF1A axis modulates the DLBCL tumor autophagy, we further detected the level of autophagy in DLBCL cells. By observing autophagy flux by confocal microscopy, SPOP deficiency was found to increase the number of autophagosomes and autolysosomes in DLBCL cells, while CHAF1A inhibition in SPOP-deficient cells completely blocked these effects (Fig. 6A, B). Lastly, TEM assays revealed a significant increase in the number of autophagic vacuoles in SPOP-deficient DLBCL cells compared with parental cells. CHAF1A knockdown could reverse the effects of SPOP deficiency (Fig. 6C). Taken together, SPOP/CHAF1A axis restricts tumor autophagy in a TFEB-dependent manner, and targeting TFEB-mediated signaling represents a useful strategy for SPOP-deficient DLBCL.

## Discussion

Intensive studies have highlighted that dysregulation of the epigenome is an essential factor during the pathogenesis of malignancies, including DLBCL [30]. As is well documented, EZH2 encodes the catalytic subunit of Polycomb repressive complex 2 (PRC2) and participates in restricting gene expression via regulating methylation of histone H3 on lysine 27 (H3K27) [31]. EZH2 is highly expressed in germinal center (GC) B cells and targeted by somatic mutations in B cell lymphomas. High-throughput sequencing have detected nealy 20% Tyr641 mutations across EZH2 in GCB-DLBCL samples [32]. DLBCLs harbouring EZH2^Y641^ mutations exhibit notably higher enrichment of trimethylated histones (H3K27me3) modifications versus DLBCLs with wild type EZH2, implicating the elevated PRC2 activity. Besides, nearly 30% of DLBCLs have the histone monomethyltransferase KMT2D mutations. Most of mutations are nonsense or frameshift mutations that could contribute to loss-of-function of KMT2D [33, 34]. KMT2D deficiency could lead to the decrease of H3K4 methylation, contributing to altered expressions of genes related to JAK-STAT signaling, cell cycle, as well as apoptosis. Furthermore, CREBBP and EP300 mutations are often mutually exclusive in DLBCLs, correlating with tumor recurrence and inferior clinical outcomes [35, 36]. These two epigenetic regulators belong to KAT3 family members of histone acetyltransferases, depending on H3K27 acetylation to function as transcriptional co-activators. Therefore, aberrant chromatin-modifying genes with mutations or abnormal expressions could potentiate lymphomagenesis via altering dysregulation of chromatin signatures. In this study, we focused on the epigenetic roles of CHAF1A in DLBCLs. Bioinformatic analysis and IHC assays consistently showed that CHAF1A expresses highly in DLBCL than normal tissues. Kaplan–Meier survival curves also indicated that DLBCLs with high CHAF1A expressions had worse OS outcomes relative to those with low CHAF1A. We further compared the differential expression levels of CHAF1A in multiple DLBCL cell lines. We utilized the lentiviruses to knock down CHAF1A expressions and found that targeting CHAF1A could significantly suppress cell growth, colony formation, and migration abilities. Meanwhile, ectopic expression of CHAF1A could restore the impaired malignant features, implicating that CHAF1A is indispensable for DLBCs aggresiveness. The subcutaneous mice model further suggested that CHAF1A could promote in vivo tumor growth, as revealed by larger tumor volumes and tumor weight. Mechanistically, we observed that E3 ligase enzyme SPOP could interact with CHAF1A and promote CHAF1A ubiquitination and ubiquitin-dependent proteasomal degradation. Aberrant DLBCL-associated SPOP mutations, like F102I or D104H, are deficient in mediating CHAF1A degradations. Low or mutated SPOP could result in accumulated CHAF1A proteins, thereby enhancing cell malignant aggressiveness. SPOP deficiency mainly depended on CHAF1A to accelerate in vivo DLBCL tumor growth. To further uncover the downstream pathways that are required for SPOP/CHAF1A axis, we found that CHAF1A mainly activated TFEB expressions. CHAF1A directly binds to the promoter region of TFEB to enhance the regional activity, as evidenced by the active chromatin indicator of H3K27ac. Subsequent analysis further revealed that CHAF1A could enhance the transcriptional activity of TFEB to promote its downstream targets, including CLEAR, CTSA, CTSD, MCOLN1, LAMP2, or ARSB. Thereby, SPOP/CHAF1A axis could restrict the expression levels of autophagy and lysosomal biogenesis-related genes. Based on the basic theory, we proposed that targeting TFEB-dependent autophagy process is effective to suppress DLBCLs with aberrant SPOP/CHAF1A axis.

Autophagy is an evolutionarily conserved physiological process during which the cellular components are recycled by lysosomes for subsequent degradation [37, 38]. This process could be induced by nutrient- and energy-limiting conditions, imbalance of reactive oxygen species (ROS), as well as endoplasmic reticulum (ER) stress [39]. Multiple aspects of biological processes are modulated by autophagy, such as cell proliferation, immune regulations, neurodegenerative disorders, or metabolic deseases [40]. In oncology, autophagy was regarded to possess essential roles to mediate resistance to targeted treatment and immunotherapy [41, 42]. As autophagy may have multi-faceted roles in cancer and is partially elucidated nowadays, development of agents that target autophagy is promosing to treat cancer [43, 44]. During the early stage of tumorigenesis, autophagy mainly suppress the accumulations of oncogenic p62 proteins, cell injury, and inflammation [45]. Therefore, autophagy may exert tumor-suppressive roles in restricting proliferation, invasion, or distal metastasis at the early stages. From the another aspect, autophagy can maintain functional mitochondria to reduce DNA damages or ROS stress, thereby enhancing the survival capacity and resistance of tumors against environmental factors. Multiple factors have contributed to the roles of autophagy on the aggressiveness of the cancers, like cancer stages, genetic phenotypes, as well as tumor microenvironment. As a master regulator of lysosomal biogenesis and autophagy, TFEB correlates tightly with multiple physiological and pathological events [46, 47]. TFEB mainly localizes to the cytoplasm under the normal situations. However, nutrient starvation could enhance TFEB nuclear translocation to exert its transcriptional activity. Previous studies have indicated that TFEB-driven autophagy is indespensible for TGF-β-induced pancreatic tumorigenesis, promoting tumor cells migration ability and in vivo metastasis [48]. Besides, TFEB could elevate PD-L1 expressions to promote immune evasion resistance to mTOR inhibition in renal cell carcinoma, highlighting the significant relationships between TFEB and immunotherapy [49]. Given that little was reported about the roles of TFEB in DLBCL, we found that CHAF1A mainly depends on activated TFEB to drive tumor progression. The TFEB-dependent autophagy is required for the oncogenic roles of CHAF1A in DLBCLs.

Although intensive researches have comprehensively described the roles of aberrant SPOP mutations or expressions on kidney cancer, prostate cancer and endometrial cancer, the specific roles of SPOP on hematologic tumors are still unclear [25, 50]. SPOP is observed to mutate in a subset of lymphoid malignancies, like DLBCLs. The lymphoid malignancies-associated SPOP mutants failed to bind to MyD88 and further restrict NF-κB activation, thus enhancing DLBCL progression. Xiaofeng Jin et al. have indicated that SPOP is a tumor suppressor in DLBCL and defective mutations in the SPOP–MyD88 binding interface may contribute to aberrant MyD88/NF-κB activation [27]. In line with the above findings, we identified that SPOP was downregulated and habours defective mutations in DLBCL, contributing to CHAF1A accumulations. Apart from MyD88/NF-κB axis, we also proposed that CHAF1A/TFEB may be the another bypass that triggers DLBCL aggressiveness induced by SPOP deficiency. In addition, Qing Shi et al. have found that cytoplasmic SPOP could bind and induce the non-degradative ubiquitination of p62, thereby decreasing p62 puncta formation and suppressing p62-dependent autophagy [24]. Apart from the identified p62/SQSTM1-dependent pathway, our study also indicated that SPOP could employ CHAF1A/TFEB axis to inhibit TFEB-dependent autophagy. Therefore, our study not only supplied novel insights on the roles of SPOP/CHAF1A axis in DLBCL tumorigenesis, but linked the mechanistic associations between SPOP and TFEB-dependent autophagy.

However, there are still some unclear problems that deserve further improvements. Firstly, the DLBCL patients samples are limited and it may take a long time to collect enough eligible patients to assess the clinical significance of SPOP/CHAF1A/TFEB axis in DLBCLs. Meanwhile, apart from SPOP-mediated ubiquitination mechanisms, we speculated that there may exist other upstream signals and epigenetic events that govern high CHAF1A expressions, including methylation, N6-methyladenosine (m6A) modifications, as well as phosphorylation at specific residues. Last of all, the in vivo efficacy of targeting TFEB on DLBCL may need more pre-clinical models to figure out.

In conclusion, our research, for the first time, highlighted the SPOP/CHAF1A ubiquitination crosstalk in the pathogenesis of DLBCL. Aberrantly high CHAF1A expressions potentiate the aggressiveness of DLBCL and indicate a inferior prognosis for patients. Deficient SPOP contributes to accumulated CHAF1A proteins, thereby sustaining tumor autophagy via induction of TFEB. Targeting TFEB is effective for DLBCL with aberrant SPOP/CHAF1A axis. These findings systematically elucidated the biological roles of SPOP/CHAF1A/TFEB pathway, endowing novel therapeutic strategies in DLBCL.

## Data Availability

The molecular experiment data generated and analyzed during the current study are available from the corresponding author with the reasonable request.

## References

[1] Ansell SM (2018). Hodgkin lymphoma: 2018 update on diagnosis, risk-stratification, and management. Am J Hematol.

[2] Graus F, Arino H, Dalmau J (2014). Paraneoplastic neurological syndromes in Hodgkin and non-Hodgkin lymphomas. Blood.

[3] Siegel RL, Miller KD, Fuchs HE, Jemal A (2022). Cancer statistics, 2022. CA Cancer J Clin.

[4] Schmitz R, Wright GW, Huang DW (2018). Genetics and pathogenesis of diffuse large B-cell lymphoma. N Engl J Med.

[5] Schmitt A, Xu W, Bucher P (2021). Dimethyl fumarate induces ferroptosis and impairs NF-kappaB/STAT3 signaling in DLBCL. Blood.

[6] Ramis-Zaldivar J, Gonzalez-Farré B, Balagué O (2020). Distinct molecular profile of IRF4-rearranged large B-cell lymphoma. Blood.

[7] Wilson WH, Wright GW, Huang DW (2021). Effect of ibrutinib with R-CHOP chemotherapy in genetic subtypes of DLBCL. Cancer Cell.

[8] Zhang J, Grubor V, Love CL (2013). Genetic heterogeneity of diffuse large B-cell lymphoma. Proc Natl Acad Sci USA.

[9] Ruppert AS, Dixon JG, Salles G (2020). International prognostic indices in diffuse large B-cell lymphoma: a comparison of IPI, R-IPI, and NCCN-IPI. Blood.

[10] Montalban C, Diaz-Lopez A, Dlouhy I (2017). Validation of the NCCN-IPI for diffuse large B-cell lymphoma (DLBCL): the addition of beta2 -microglobulin yields a more accurate GELTAMO-IPI. Br J Haematol.

[11] Prochazka KT, Melchardt T, Posch F (2016). NCCN-IPI score-independent prognostic potential of pretreatment uric acid levels for clinical outcome of diffuse large B-cell lymphoma patients. Br J Cancer.

[12] Biccler J, Eloranta S, de Nully BP (2018). Simplicity at the cost of predictive accuracy in diffuse large B-cell lymphoma: a critical assessment of the R-IPI, IPI, and NCCN-IPI. Cancer Med.

[13] Fontan L, Goldstein R, Casalena G (2021). Identification of MALT1 feedback mechanisms enables rational design of potent antilymphoma regimens for ABC-DLBCL. Blood.

[14] Li W, Gupta SK, Han W (2019). Targeting MYC activity in double-hit lymphoma with MYC and BCL2 and/or BCL6 rearrangements with epigenetic bromodomain inhibitors. J Hematol Oncol.

[15] Bakhshi TJ, Georgel PT (2020). Genetic and epigenetic determinants of diffuse large B-cell lymphoma. Blood Cancer J.

[16] Serganova I, Chakraborty S, Yamshon S (2021). Epigenetic, metabolic, and immune crosstalk in germinal-center-derived B-cell lymphomas: unveiling new vulnerabilities for rational combination therapies. Front Cell Dev Biol.

[17] Liu T, Wei J, Jiang C (2017). CHAF1A, the largest subunit of the chromatin assembly factor 1 complex, regulates the growth of H1299 human non-small cell lung cancer cells by inducing G0/G1 cell cycle arrest. Exp Ther Med.

[18] Barbieri E, De Preter K, Capasso M (2014). Histone chaperone CHAF1A inhibits differentiation and promotes aggressive neuroblastoma. Cancer Res.

[19] Shen J, Liu X, Zhou M (2020). CHAF1A overexpression in human retinoblastoma promotes cell proliferation and suppresses apoptosis. J BUON.

[20] Xu M, Jia Y, Liu Z (2016). Chromatin assembly factor 1, subunit A (P150) facilitates cell proliferation in human hepatocellular carcinoma. Onco Targets Ther.

[21] Song Y, Xu Y, Pan C (2020). The emerging role of SPOP protein in tumorigenesis and cancer therapy. Mol Cancer.

[22] Zhang J, Bu X, Wang H (2018). Cyclin D-CDK4 kinase destabilizes PD-L1 via cullin 3-SPOP to control cancer immune surveillance. Nature.

[23] Bernasocchi T, El Tekle G, Bolis M (2021). Dual functions of SPOP and ERG dictate androgen therapy responses in prostate cancer. Nat Commun.

[24] Shi Q, Jin X, Zhang P (2022). SPOP mutations promote p62/SQSTM1-dependent autophagy and Nrf2 activation in prostate cancer. Cell Death Differ.

[25] Wang Z, Song Y, Ye M (2020). The diverse roles of SPOP in prostate cancer and kidney cancer. Nat Rev Urol.

[26] Wang L, Lin M, Chu M (2020). SPOP promotes ubiquitination and degradation of LATS1 to enhance kidney cancer progression. EBioMedicine.

[27] Jin X, Shi Q, Li Q (2020). CRL3-SPOP ubiquitin ligase complex suppresses the growth of diffuse large B-cell lymphoma by negatively regulating the MyD88/NF-kappaB signaling. Leukemia.

[28] Oughtred R, Stark C, Breitkreutz BJ (2019). The BioGRID interaction database: 2019 update. Nucleic Acids Res.

[29] Oughtred R, Rust J, Chang C (2021). The BioGRID database: a comprehensive biomedical resource of curated protein, genetic, and chemical interactions. Protein Sci.

[30] Kuhnl A, Cunningham D, Chau I (2017). Beyond genomics—targeting the epigenome in diffuse large B-cell lymphoma. Cancer Treat Rev.

[31] McCabe MT, Ott HM, Ganji G (2012). EZH2 inhibition as a therapeutic strategy for lymphoma with EZH2-activating mutations. Nature.

[32] Morin RD, Johnson NA, Severson TM (2010). Somatic mutations altering EZH2 (Tyr641) in follicular and diffuse large B-cell lymphomas of germinal-center origin. Nat Genet.

[33] Zhang J, Dominguez-Sola D, Hussein S (2015). Disruption of KMT2D perturbs germinal center B cell development and promotes lymphomagenesis. Nat Med.

[34] Xu-Monette ZY, Wei L, Fang X (2022). Genetic subtyping and phenotypic characterization of the immune microenvironment and MYC/BCL2 double expression reveal heterogeneity in diffuse large B-cell lymphoma. Clin Cancer Res.

[35] Huang YH, Cai K, Xu PP (2021). CREBBP/EP300 mutations promoted tumor progression in diffuse large B-cell lymphoma through altering tumor-associated macrophage polarization via FBXW7-NOTCH-CCL2/CSF1 axis. Signal Transduct Target Ther.

[36] Meyer SN, Scuoppo C, Vlasevska S (2019). Unique and shared epigenetic programs of the CREBBP and EP300 acetyltransferases in germinal center B cells reveal targetable dependencies in lymphoma. Immunity.

[37] Kuma A, Komatsu M, Mizushima N (2017). Autophagy-monitoring and autophagy-deficient mice. Autophagy.

[38] Onorati AV, Dyczynski M, Ojha R, Amaravadi RK (2018). Targeting autophagy in cancer. Cancer.

[39] Klionsky DJ, Petroni G, Amaravadi RK (2021). Autophagy in major human diseases. EMBO J.

[40] Mizushima N, Levine B (2020). Autophagy in human diseases. N Engl J Med.

[41] Jiang GM, Tan Y, Wang H (2019). The relationship between autophagy and the immune system and its applications for tumor immunotherapy. Mol Cancer.

[42] Xia H, Green DR, Zou W (2021). Autophagy in tumour immunity and therapy. Nat Rev Cancer.

[43] Mowers EE, Sharifi MN, Macleod KF (2018). Functions of autophagy in the tumor microenvironment and cancer metastasis. FEBS J.

[44] Dikic I, Elazar Z (2018). Mechanism and medical implications of mammalian autophagy. Nat Rev Mol Cell Biol.

[45] Jiang T, Harder B, Rojo de la Vega M (2015). p62 links autophagy and Nrf2 signaling. Free Radic Biol Med.

[46] Medina DL, Di Paola S, Peluso I (2015). Lysosomal calcium signalling regulates autophagy through calcineurin and TFEB. Nat Cell Biol.

[47] Nnah IC, Wang B, Saqcena C (2019). TFEB-driven endocytosis coordinates MTORC1 signaling and autophagy. Autophagy.

[48] He R, Wang M, Zhao C (2019). TFEB-driven autophagy potentiates TGF-beta induced migration in pancreatic cancer cells. J Exp Clin Cancer Res.

[49] Zhang C, Duan Y, Xia M (2019). TFEB mediates immune evasion and resistance to mTOR inhibition of renal cell carcinoma via induction of PD-L1. Clin Cancer Res.

[50] Mani RS (2014). The emerging role of speckle-type POZ protein (SPOP) in cancer development. Drug Discov Today.

